# The Effect of Specific Treadmill Protocol on Aerobic Performance Parameters in Flat-Terrain-Trained Athletes

**DOI:** 10.3390/life15040569

**Published:** 2025-04-01

**Authors:** Ming-Chang Tsai, Edward Lin, Scott Thomas

**Affiliations:** 1 Canadian Sport Institute Pacific, Victoria, BC V9E 2C5, Canada; 2 Toronto Western Hospital, Toronto, ON M5T 2S8, Canada; edward.lin@uhn.ca; 3 Graduate Department of Exercise Sciences, Faculty of Kinesiology and Physical Education, University of Toronto, Toronto, ON M5S 2W6, Canada; scott.thomas@utoronto.ca

**Keywords:** testing, exercise, training, *VO*_2*max*_, inclined

## Abstract

This study examined the differences in physiological, metabolic and running dynamics responses between level and inclined treadmill protocols and their implications for accurately determining training intensities. Twenty-three healthy, active adults (18 male and 5 female) from 25 to 59 years old (age: 42.7 years, height: 1.77 m, body mass: 71.9 kg, $VO_{2max}$: 54.3 mL·${\mathrm{kg}}^{-1}$·$\min^{-1}$) completed both protocols. Physiological markers (gas exchange threshold (GET), respiratory compensation point (RCP), $VO_{2max}$), metabolic variables (HR, $VO_{2}$, $VCO_{2}$, RER, VE, speed) and running dynamic variables (running economy (RE), stride length (SL), ground contact time (GCT), cadence) were measured and matched for the external work rate at each stage. The data were analyzed using one-way repeated measures ANOVA with Tukey’s post hoc procedure. No significant differences were observed in the physiological markers for the inclined and flat protocols across all the intensities. However, the metabolic variables showed significant differences (*p* = 0.0333 to <0.0001) between the inclined and flat protocols at higher intensities. The RE was consistently improved in the flat protocol compared with the inclined protocol, with significant differences observed at the high-intensity stages (*p* = 0.0232 to <0.0001). While the physiological markers remained unaffected, metabolic responses and running kinematics differed significantly between the protocols. These results highlight that training intensity zones derived from inclined protocols may not be appropriate for flat terrain training, underlining the importance of testing specificity in athlete preparation.

## 1. Introduction

Aerobic capacity is often assessed with a treadmill test of maximum oxygen uptake ($VO_{2max}$). An individual’s $VO_{2max}$, along with other physiological variables measured during this test, is an important clinical and performance indicator [1,2]. For athletes, it is often used to prescribe training and performance intensities, as recommended by the American College of Sports Medicine [3] and observed in many recent studies [4,5]. Therefore, the results of treadmill tests play a crucial role in the preparation of an athlete’s competition.

An early treadmill protocol was developed by Taylor et al. in 1955, which involved a discontinuous test that was conducted over multiple days while increasing the treadmill incline (grade) and keeping the speed constant. Subsequent protocols, such as the Balke, Bruce and the World Health Organization (WHO) protocols [6], also used an increasing treadmill grade and have been widely used with patients and athletes. Many concerns were raised about their application [7,8], as the increments in intensity and test duration are not suitable for all groups and all require steep running. The current standard in treadmill exercise protocols is to increase the intensity through increases in grade, forcing the participant to run at higher inclines as the intensity reaches competitive intensity equivalents. However, training and competition occur on both level and inclined terrains, and testing conditions were shown to closely simulate the specific sport practiced by the athlete [9]. Thus, a single testing mode may not accurately determine the training and competition intensity zones. However, there is a lack of studies that match the external work rate between flat and inclined protocols, which is critical to isolate the effect of the incline on metabolic and physiological responses. Previous research often compared protocols without standardizing kinetic energy requirements, potentially conferring biased results for the training description.

Despite the prevalence of inclined treadmill protocols, recent research explored more individualized and self-paced approaches to improve the ecological validity of testing. For example, a study found that self-paced intermittent protocols on non-motorized treadmills can reliably mimic the fluctuating intensity of real-world athletic competitions [10]. Additionally, individualized treadmill protocols, where participants control the speed and incline based on their preferences, were shown to yield higher $VO_{2max}$ values compared with standardized tests, suggesting a closer reflection of true aerobic capacity [11]. Furthermore, a four-minute self-paced running time trial on a treadmill was validated as a reliable method for a $VO_{2max}$ assessment [12], offering a fixed-duration, athlete-centered approach. However, these approaches have not addressed whether the commonly used inclined testing protocols appropriately reflect the metabolic demands of flat terrain running, particularly when the training zone prescription is based on data obtained under non-specific conditions.

Despite the obvious problem, there were a few studies that attempted to optimize treadmill test protocols. A group of researchers suggested a new $VO_{2max}$ protocol that allows participants to self-pace within stages of the test while maintaining an incremental procedure [13]. Others explored the effects of manipulating the frequency of data acquisition on the achievement of maximum oxygen consumption [14] and examined the implications of altering the duration of stages on various physiological variables during incremental testing protocols [15]. However, little has been done to address the issue that incremental inclined tests do not provide an opportunity to assess high-intensity performance on a level surface.

Running on flat terrain compared with inclined terrain involves differences in running economy; running dynamics; and, ultimately, $VO_{2max}$. Multiple studies found that increasing the grade resulted in an increased energetic cost of running, and therefore, a lower running economy [16,17,18]. A higher stride frequency and reduced stride length were observed in treadmill running on an incline [19], which has been known to increase the metabolic cost of running at given speeds [20]. An increased peak oxygen deficit was also recorded in incline running compared with flat running [21]. The authors attributed the increased peak oxygen deficit to the greater leg muscle activation seen in incline running, which may have resulted in a decreased running efficiency. Ground contact times during running may play a role in this, as many studies indicated that a reduced ground contact time relates to a decreased metabolic cost [22,23]. These studies that compared the biomechanics behind the different running types indicate the potential for incline running to cause earlier and greater leg fatigue, which decreases the maximal oxygen uptake. There are contradictory observations about the effect of flat and inclined protocols on $VO_{2max}$. Some studies indicated differences in the $VO_{2max}$ achieved between incline and flat protocols [24,25,26], while other studies found none [27,28]. Alternative markers of intensity, such as heart rate, respiratory exchange ratio and maximum ventilation, may be higher with inclined running, while protocol effects on the gas exchange threshold have not been characterized [27]. However, none of these studies matched speeds between stages in inclined and flat protocols and did not compare the metabolic data at each stage.

The purpose of this study was to compare physiological markers (gas exchange threshold, respiratory compensation point and $VO_{2max}$) and metabolic variables ($VO_{2}$, $VCO_{2}$, VE, RER and HR) at a common external work rate between the flat and inclined treadmill protocols. By standardizing the external workload between protocols, this study sought to fill a gap in current research and provide evidence for the need to align testing modalities with athletes’ actual training environments.

## 2. Materials and Methods

### 2.1. Experimental Approach to the Problem

Following familiarization with the study protocols and procedures, this study utilized a repeated-measures crossover design, where each participant completed both the flat and inclined treadmill protocols in a randomized order to minimize order effects. The participants attended one session in the laboratory to perform two ramp tests (randomized order), with either inclined or flat protocols for the determination of the aerobic parameters: $VO_{2max}$, gas exchange threshold (GET) and respiratory compensation point (RCP), with a 30–45 min rest between the tests. This rest period was selected based on Hinckson and Hopkins (2005), who demonstrated that 30 min was sufficient to maintain test–retest reliability in time-to-exhaustion tests, with a coefficient of variation below 5% and no significant performance decrease observed in the second trial, indicating effective recovery between tests [29]. The design ensured that each participant served as their own control, which enabled a direct comparison between the testing conditions. All exercise tests were conducted using a calibrated treadmill (Woodway Path, Waukesha, WI, USA) that was capable of adjusting with 0.1% grade and 0.045 m·$s^{-1}$ increments. The running dynamics variables (ground contact time (GCT), cadence) were measured second by second with a Garmin Forerunner 620 (Olathe, KS, USA). Metabolic variables (RER, heart rate (HR), VE, $VO_{2}$, $VCO_{2}$) were measured breath-by-breath throughout the test from a metabolic analyzer (Metamax^®^ 3B, Leipzig, Germany), and the $VO_{2max}$ was determined as the highest average $VO_{2}$ over a 20 s period. The GET and RCP were estimated using the V-slope method [30] by identifying breakpoints in the $VO_{2}$ vs. $VCO_{2}$ relationships. Given the variability in threshold identification, two independent researchers reviewed and confirmed the inflection points. In cases of ambiguity, a third reviewer was consulted. The running economy was calculated as the energetic cost of running in mL·$O_{2}\cdot$${\mathrm{km}}^{-1}$.

### 2.2. Subjects

The participants who were members of local running clubs were recruited through postings and word of mouth to take part in this study. The candidate participants were informed about the study aims, procedures and risks associated with the tests, and all participants gave their written and informed consent. This study was approved by the University of Toronto Review Ethics Board and was conducted in accordance with the Declaration of Helsinki. The participants were asked to refrain from participating in strenuous physical activity in the 24 h prior to the test and avoid caffeine and alcohol consumption 3 h before reporting to the laboratory. Twenty-three healthy, athletic adults (18 male and 5 female) from 25 to 59 years old (Table 1) participated in this study.

### 2.3. Procedures

The participants were screened using the PAR-Q+ and an athlete consent form in person. The PAR-Q+ (Physical Activity Readiness Questionnaire) was used to determine the safety and possible risks for an individual to begin the exercise program [31]. The questionnaire was used in several studies that involved maximum efforts [32,33,34]. Once written consent was obtained, subjects were familiarized with all the protocols and procedures. In addition, the participants were instructed on how to immediately signal to the tester whether they experienced discomfort, dizziness or any adverse symptoms that required the test to be stopped. A safety clip connected to the treadmill’s emergency stop mechanism was worn by each participant throughout testing. Trained personnel were present at all times to ensure an immediate response to any emergency situation.

This study included twenty-three trained runners (18 males, 5 females), which ensured a homogeneous athletic population for assessing the aerobic capacity and running dynamics.

Inclusion criteria:

- Age 18+;
- Engagement in regular aerobic activity (≥3 days per week for at least 30 min per session) for a minimum of six months;
- Familiarity with treadmill running.

Exclusion criterion:

- Failure to meet the PAR-Q+ physical activity readiness screening.

A priori power analysis was conducted using G*Power (Version 3.1.9.6, Heinrich Heine University Dusseldorf, Dusseldorf, Germany) to determine the required sample size. The analysis was based on prior studies that examined $VO_{2max}$, metabolic variables and running kinematics across treadmill protocols. The following parameters were used: effect size (F) = 0.30, α = 0.05, power (1 −β) = 0.80 and a within-subject correlation of 0.5 (based on similar treadmill-based studies). The analysis indicated that a minimum sample size of 20 participants was required to detect significant differences between the protocols. To account for potential dropouts and ensure statistical power, 23 participants were recruited.

The participants warmed up for 5–10 min on a treadmill at a self-selected comfortable speed, which was subsequently used as their starting speed after 5–10 min of rest. Each treadmill test began with 2 stages of 2 min duration at lower intensities (i.e., 1% and 3% grade or incline-matched speed), and then the intensity was increased (either 2% grade or incline-matched speed) every minute until volitional exhaustion or the participants signaled to the tester that they could not complete the next 30 s period. A 1% grade was chosen as the starting level in both protocols since it most accurately represents the energetic costs of outdoor running [35]. The treadmill speeds in the flat protocols were determined using energy-matching equations at each distinct stage (see below). Strong verbal encouragement was provided to elicit full effort. To ensure consistency across the tests, the same researcher provided standardized verbal encouragement to all the participants. Pre-determined phrases were used at consistent time points throughout the test to maintain uniform motivation. Since this study utilized a within-subjects design, each participant served as their own control, which mitigated potential variability in encouragement between the trials. To further minimize the bias, the researcher followed a scripted encouragement protocol and avoided any unintentional cues that could influence the performance.

The attainment of $VO_{2max}$ was confirmed by the achievement of a plateau in $VO_{2}$ (increase of <2 mL·${\mathrm{kg}}^{-1}\cdot$$\min^{-1}$ despite an increase in workload) [36,37], RER values above 1.20 and a heart rate within 5 bpm of the age-predicted maximum. For all the participants, at least two of the three criteria were met. Expired breath-by-breath gas exchange data were collected continuously from a metabolic analyzer (Metamax^®^ 3B, Leipzig, Germany). The second test was performed on the same day after 30 min of rest.

### 2.4. Speed Calculations

The speed for each stage in the flat protocol was calculated using kinetic equations to ensure the stages between the flat and inclined protocols were matched in kinetic energy. Kinetic energy considers the mass of the individual, as well as their speed, and is represented by the following equation:

(1) $$E=\frac{1}{2}mv^{2}$$

where *E* represents the energy in Joules, *m* represents the mass in kilograms and *v* represents the speed in m·$s^{-1}$. To increase the kinetic energy expended, two methods were employed: (1) the grade or incline of the treadmill was increased (which increased the effective mass due to gravity) while the speed remained constant, and (2) the speed was increased as the grade remained constant. These two methods were the premises of the inclined and flat protocols we designed for this study. To start, the kinetic energy on an incline is represented by the following equation:

(2) $$E=\frac{1}{2}Mv_{inc}^{2}$$

where *M* represents the mass in kilograms (considering the incline) and $v_{inc}$ represents the speed on the incline in m·$s^{-1}$. Due to the fact the participant was running on an incline, the mass was affected by gravity and the angle of inclination. In the protocol, we used the grade to describe the level of the incline. The grade refers to the rise over run of the treadmill and is expressed as a percentage (units of rise per 100 units of run). Taking into account the effect of gravity, we used the following equation to determine the effective mass *M*:

(3) M=m+m·g·sin(x)

where *m* represents the body mass in kilograms, *g* represents the gravitational acceleration (9.81 m·$s^{-2}$) and *x* represents the inclination in degrees. Substituting Equation (3) into Equation (2) results in

(4) $$E=\frac{1}{2}\left(m+m\dot{g}\cdot sin\left(x\right)\right)\cdot v_{inc}^{2}$$

The kinetic energy expended during the flat protocol is represented by a similar equation but without having to account for gravity and the inclination:

(5) $$E=\frac{1}{2}mv_{flat}^{2}$$

where $v_{flat}$ represents the speed during the flat protocol in m·$s^{-1}$. To determine the speeds for the flat protocol, the two energy Equations (4) and (5) were combined:

(6) $$\frac{1}{2}\left(m+m\dot{g}\cdot sin\left(x\right)\right)\cdot v_{inc}^{2}=\frac{1}{2}mv_{flat}^{2}$$

By isolating for $v_{flat}$ the resulting equation is

(7) $$v_{flat}=\sqrt{1+g\cdot sin(x)}\cdot v_{inc}$$

Using this equation, we could determine the speeds for the participants at each stage of the flat protocol from the grade of the inclined protocol stage and initial speed the participants selected.

### 2.5. Statistical Analysis

The individual stage oxygen uptake threshold, running dynamics and metabolic variables for the inclined and flat protocols were compared using a two-way repeated measures analysis of variance mixed model in R (version 4.4.2, Vienna, Austria), with Tukey’s HSD post hoc procedure used to control for type I errors in making multiple comparisons and determine the significant differences between the variables. Significance was set at *p* < 0.05, and the *p*-values are reported in the results. Prior to the analysis, data were tested for normality using the Shapiro–Wilk test, and the sphericity was assessed with Maulchly’s test. When the sphericity assumptions were violated, a Greenhouse–Geisser correction was applied. The residual plots were all inspected to confirm the homogeneity of variance to ensure that the presuppositions for using parametric analysis were satisfied.

## 3. Results

**Figure 1:**
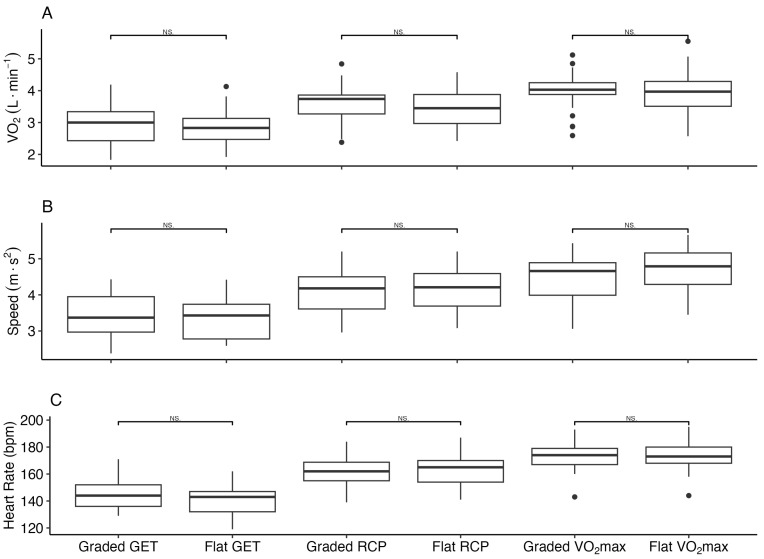
(**A**) $VO_{2}$, (**B**) speed and (**C**) HR corresponding to the attained threshold markers GET, RCP and $VO_{2max}$ during the horizontal (flat) and inclined (graded) treadmill running by fit young adults (n = 23). NS represents non-significant; GET is the gas exchange threshold; RCP is the respiratory compensation point; $VO_{2max}$ is the maximum oxygen consumption (all threshold values expressed in L·$\min^{-1}$).

There was no difference in the markers $VO_{2}$, speed and HR between the grade and flat protocols at the threshold points GET, RCP and $VO_{2max}$, as represented in Figure 1.

**Figure 2:**
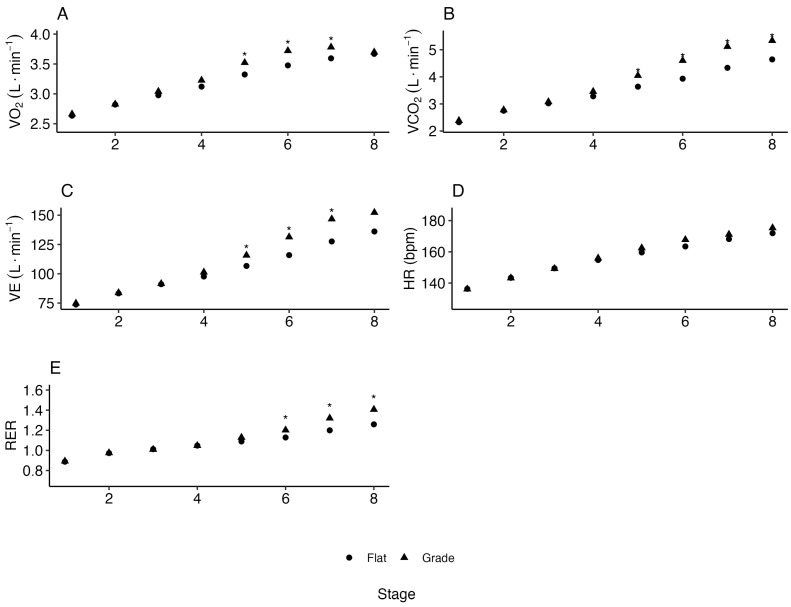
(**A**) Oxygen consumption ($VO_{2}$, L·$\min^{-1}$); (**B**) carbon dioxide output ($VCO_{2}$, L·$\min^{-1}$); (**C**) minute ventilation (VE, L·$\min^{-1}$); (**D**) heart rate (HR, bpm); and (**E**) respiratory exchange ratio (RER) at each stage during horizontal (flat) and inclined (graded) treadmill running. * represents *p* < 0.05.

There were significant differences between the inclined and flat protocols for the metabolic variable minute ventilation (VE). These differences were observed in the latter stages of the protocols, with higher values observed in stages 6–8 (Figure 2B). There were no significant differences in the $VO_{2}$ between the grade and flat protocols at the GET: 2.92 L·$\min^{-1}$ vs. 2.84 L·$\min^{-1}$ (*p* = 1), RCP: 3.62 L·$\min^{-1}$ vs. 3.42 L·$\min^{-1}$ (*p* = 1) and $VO_{2max}$: 3.99 L·$\min^{-1}$ vs. 3.90 L·$\min^{-1}$ (*p* = 1). Alternative treadmill protocols (grade, flat) produced no significant differences in the running speed at the GET: 3.45 m·$s^{-1}$ vs. 3.38 m·$s^{-1}$ (*p* = 1), RCP: 4.10 m·$s^{-1}$ vs. 4.16 (*p* = 1) and $VO_{2max}$: 4.48 m·$s^{-1}$ vs. 3.70 m·$s^{-1}$ (*p* = 1). There was no significant difference in the HR between the grade and flat protocols at the GET: 144.5 bpm vs. 140.8 bpm (*p* = 0.877), RCP: 162.4 bpm vs. 162.9 bpm (*p* = 1) and $VO_{2max}$: 172.9 vs. 173.9 bpm (*p* = 1).

**Figure 3:**
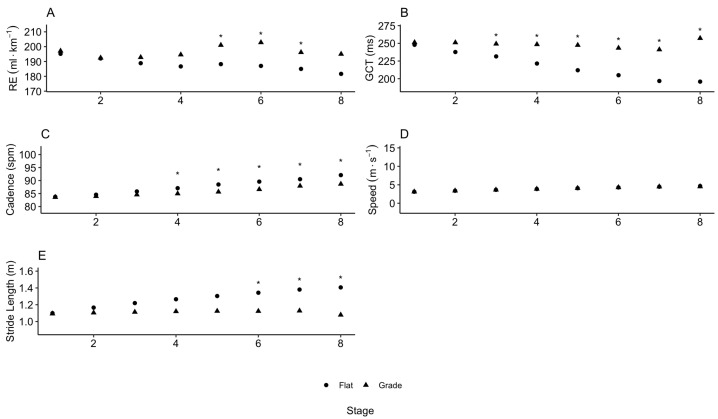
(**A**) Running economy (RE, mL·${\mathrm{km}}^{-1}$); (**B**) ground contact time (GCT, msec); (**C**) cadence (strides·$\min^{-1}$); (**D**) speed (m·$s^{-1}$); and (**E**) stride length (SL, m) at each stage during horizontal (flat) and inclined (graded) treadmill running. * represents *p* < 0.05.

Figure 3 represents a comparison of the running dynamics variables (RE, cadence, stride length (SL) and ground contact time (GCT)) between the protocols at each stage, which also produced meaningful results. The ground contact time remained no different in the flat protocols compared with the inclined protocol through the first three stages and significantly less in stages 4–8 (*p* < 0.001). The minute ventilation showed no difference between the two protocols for the beginning-to-mid stages of the test but were higher in the last three stages in the graded compared with flat protocols (*p*-values were 0.01, 0.001 and 0.0489). The ground contact time remained relatively constant throughout the stages in the inclined protocol, while it gradually increased from stage to stage in the flat protocols. Like the stride length, the ground contact time remained relatively constant throughout the stages in the inclined protocol, while it steadily decreased from stage to stage in the flat protocol. In terms of the testing duration, all the participants performed a longer test on the flat protocol compared with the inclined one. The inclined test lasted on average 9.4 ± 1.0 min and up to stage 7.5, while the flat test lasted on average 10.9 ± 1.0 min and up to stage 9.1.

Table 2 presents a comparative analysis of key physiological, metabolic and running dynamics variables across the flat and inclined treadmill protocols. While the $VO_{2max}$ and heart rate showed no significant differences, key metabolic markers, such as carbon dioxide output ($VCO_{2}$) and minute ventilation (VE), were significantly elevated in the inclined protocol. The running economy was reduced with incline, which reflected the increased energy cost of uphill running. Additionally, the stride length decreased and the cadence and ground contact time increased in the inclined protocol, which reinforced the biomechanical adjustments necessary for incline running. These findings emphasize the biomechanical and metabolic distinctions between training modalities and the importance of specificity in testing and training prescriptions.

## 4. Discussion

The main finding of this study was that the inclined and flat treadmill protocols elicited different responses in certain metabolic variables. Significant differences in the RER, VE, HR, $VO_{2}$ and $VCO_{2}$ were observed between the protocols near the mid-to-end stages of the tests. However, no significant differences in the $VO_{2max}$ were found between the protocols in this sample of healthy, competitive runners. These results suggest that even at matched intensities, the runners responded differently to the flat and inclined protocols. To our knowledge, this is one of the first studies to match the external work rate when comparing inclined and flat protocols, which allowed for an unbiased assessment of the metabolic demands.

The differences observed in the metabolic variables between inclined and flat protocols can be explained by several physiological mechanisms. First, inclined running requires greater activation of lower limb extensor muscles, particularly the quadriceps and calf muscles, to overcome gravity. This increased muscle recruitment, combined with a higher proportion of concentric muscle actions, leads to greater oxygen demand and higher minute ventilation at similar external workloads [21]. Second, biomechanical adjustments, such as a shorter stride length and longer ground contact time, during inclined running have been associated with reduced elastic energy storage and utilization, resulting in less efficient locomotion and increased metabolic cost [20]. These factors collectively contributed to the elevated metabolic responses observed in the inclined protocol compared with the flat protocol, despite matching for the external work rate. These findings are consistent with previous studies demonstrating greater leg muscle activation and metabolic cost during inclined running [22,23].

While no difference in the $VO_{2max}$ was observed, the $VO_{2}$ throughout the stages and resulting running economy were significantly different between the protocols. This is consistent with research that showed that exercise intensity can modulate metabolic responses, where higher intensities typically elevate cardiorespiratory demand and alter the running economy [38]. These findings are particularly relevant for track athletes, as previous studies showed that optimizing the intensity and economy is critical for enhancing the performance in middle- and long-distance running events [39]. These two variables are critical for determining training zone intensities.

This study also highlighted the importance of running kinematics in improving performance. The significant differences in running kinematics between the two protocols suggest that applying the stride length and cadence data from the inclined protocol to flat-level running competitions would be misleading. As runners fatigue, they stray away from their optimal stride length/cadence [20] or even into sometimes running with less economical patterns [40], which was shown to impact the efficiency at varying intensities [41]. Studies on track athletes demonstrated that maintaining optimal kinematic patterns, including the stride length and contact time, is associated with superior running economy and performance outcomes [42]. Feedback from the flat protocol could help runners optimize their running mechanics and reduce their aerobic demand [43].

The observed differences in the metabolic responses during the high-intensity running on a grade or flat surface align with previous research that reported greater values for maximum ventilation and RER with flat running [27]. The results differ from those of Kasch et al. (1976), who found no differences in metabolic variables between protocols [28]. However, studies indicated that the magnitude of metabolic changes may be intensity-dependent, with greater shifts occurring at or above the respiratory compensation point [44]. The contradictions in their findings may be due to differences in the protocol design, such as intensity matching and rate of inclined increase. In contrast, the present study matched the intensity and increased the grade more rapidly, which likely contributed to the observed differences in the metabolic responses.

In terms of training zone prescriptions, the two protocols produced different recommended training intensities. For example, a prescription to run at the RCP based on the inclined protocol would result in a 6% higher $VO_{2}$ compared with the flat protocol. Additionally, the $VO_{2}$ was greater from stage 4 to stage 8 in the inclined protocol compared with the flat protocol, and the HR was greater from stages 6 to 8 in the inclined protocol. This highlights the importance of matching the training conditions to effectively prescribe training zones. This further supports the notion that training adaptations are intensity-specific, and the protocol choice may affect both the short-term physiological responses and long-term adaptations [45]. The inclined protocol generally underestimates the training zones for flat running. These findings are consistent with previous research that showed that inclined running elicits greater cardiovascular and metabolic demands compared with flat running, reinforcing the need to account for terrain when prescribing the training intensity [18]. Similarly, Freund et al. (1986) observed that participants who trained on inclined terrain experienced significant increases in $VO_{2max}$, ventilation and treadmill time to exhaustion when tested on an inclined protocol compared with a horizontal protocol, emphasizing the specificity of training adaptations to the testing conditions [46].

The cost of running was higher in the inclined protocol compared with the flat protocol, suggesting a greater running economy in flat running, which is consistent with the previous literature [16,17,18], which attributes improved running economy in flat running to the stretch-shortening cycle [16] and the better utilization of muscle tendon units in the lower extremities [47]. In contrast, incline running reduces the storage of elastic energy in the muscle tendon units on landing and subsequent release of that energy on take-off [48,49]. This underutilization increases the cost of running and decreases the running economy. In terms of eccentric/concentric phases, the concentric phase is increased during incline running [16]. The concentric phase results in a 3–5× greater energy expenditure in comparison with the eccentric phase [49].

In terms of kinematics, a lower ground contact time and greater stride length are correlated with more economical running [20,22,23,40]. These findings support the idea that optimizing the cadence and stride length can lead to a reduction in submaximal oxygen consumption [50] and improve the running economy [51]. Similarly, recent studies focused on female runners confirmed that training interventions targeting stride parameters can result in significant gains in performance and economy [52].

### 4.1. Limitations

Several limitations of this study should be noted. First, the sample consisted primarily of track athletes, which may have made them more effective at the flat protocol. A more diverse sample of track and cross-country runners could provide a better representation of the two protocols.

Second, the small number of female participants (n = 5) prevented sex-based analysis, which limited our ability to explore the potential differences in physiological or biomechanical responses between male and female runners. Prior research indicates possible sex differences in running economy and metabolic responses, which may warrant investigation in future studies with larger, sex-balanced samples. Additionally, while all participants were aerobically trained, detailed training load or competitive level data were not collected, which may influence the generalizability of the findings. Future research should consider stratifying participants based on sex, training volume and competitive level to elucidate how these variables may interact with treadmill protocol responses.

Third, we did not collect detailed data on participants’ specific sports backgrounds or weekly training volume. While all participants met the inclusion criteria for regular aerobic activity and treadmill familiarity, variation in the individual training loads may have influence the responses to the treadmill protocols. Future studies should consider incorporating weekly mileage, training intensity and specific sport history to further refine the applicability of treadmill-based assessments.

Finally, we did not measure the training status or adaptations to different intensity zones over time. Future studies should examine how these protocols influence long-term training outcomes across various intensities [53].

### 4.2. Practical Applications and Future Recommendations

The findings of this study have several practical implications for both athletes and practitioners. First, given the observed differences in the metabolic and running dynamics responses between the flat and inclined treadmill protocols, we recommend that testing protocols be selected to match the specific demands of the athlete’s competition terrain. For athletes preparing for flat-ground events (e.g., track races, road running), flat treadmill protocols are more appropriate to ensure the accurate determination of training intensities, running economy and mechanical efficiency. Conversely, for athletes specializing in hill running or mountainous events, inclined protocols may offer more ecologically valid data.

Furthermore, the significant differences in running kinematics, such as stride length, cadence and ground contact time between the protocols suggest that feedback derived from terrain-specific protocols may assist coaches in designing training interventions that target optimal running mechanics.

From a research perspective, future studies should consider stratifying samples by sex, training status and age to explore potential subgroup differences, as well as incorporating longitudinal designs to assess how protocol-specific training prescriptions influence performance outcomes over time. Additionally, investigating the application of individualized or self-paced treadmill protocols in relation to terrain specificity could further refine current the testing practices.

## 5. Conclusions

The findings strongly suggest that testing protocols should be specific to the athlete’s training and competition modality. There were obvious differences in the physiological and metabolic responses to the separate protocols, as well as different running kinematics. These results directly answer this study’s goal by demonstrating that although key physiological markers, such as the $VO_{2max}$, did not differ significantly, the metabolic variables and running kinematics differed substantially, particularly at higher intensities. This implies that using inclined protocols to prescribe training zones for flat running may lead to inaccurate intensity recommendations. Utilizing the correct protocol will yield more accurate metrics, which are essential in an athlete’s preparation. At a time where analytics are at the forefront of performance optimization, testing procedures must be properly administered.

## Figures and Tables

**Table 1:** Subject characteristics.

	Male	Female
Age (years)	43.9 ± 5.7	33.5 ± 3.7
Height (m)	1.80 ± 0.09	1.64 ± 0.08
Body mass (kg)	75.0 ± 6.6	60.9 ± 4.2
$VO_{2max}$ (mL of $O_{2}\cdot$$\min^{-1}\cdot$${\mathrm{kg}}^{-1}$)	57.6 ± 5.7	47.6 ± 5.7

**Table 2:** Summary of key variables across flat and inclined protocols.

Variable	Flat Protocol	Inclined Protocol	Significance (*p*-Value)
$VO_{2}$ (L·$\min^{-1}$)	3.90 ± 0.5	3.99 ± 0.5	1.000
$VCO_{2}$ (L·$\min^{-1}$)	3.42 ± 0.4	3.62 ± 0.4	0.0489
VE (L·$\min^{-1}$)	92.5 ± 8.7	101.4 ± 9.1	0.010
HR (L·$\min^{-1}$)	173.9 ± 5.3	172.9 ± 5.5	1.000
Running economy (mL·${\mathrm{km}}^{-1}$)	185 ± 10	200 ± 11	0.0232
Stride length (m)	1.35 ± 0.1	1.28 ± 0.1	0.001
Cadence (steps·$\min^{-1}$)	1.35 ± 0.1	1.28 ± 0.1	0.001
Ground contact time (ms)	225 ± 10	238 ± 11	0.001

## Data Availability

The data used in this study were shared by the University of Toronto Track Club Masters and are not available for further sharing.
